# Effects of smoking and smoking abstinence on spatial vision in chronic heavy smokers

**DOI:** 10.1038/s41598-017-01877-z

**Published:** 2017-05-10

**Authors:** Thiago Monteiro de Paiva Fernandes, Natalia Leandro de Almeida, Natanael Antonio dos Santos

**Affiliations:** 1 Cognitive Neuroscience and Behavior Program, Federal University of Paraiba, Joao Pessoa, Brazil; 2 Perception, Neuroscience and Behavior Laboratory, Federal University of Paraiba, João Pessoa, Brazil

## Abstract

Cigarette smoke is a complex chemical mixture, involving health-damaging components such as carbon monoxide, ammonia, pyridine, toluene and nicotine. While cognitive functions have been well documented in heavy smokers, spatial vision has been less characterized. In the article, we investigated smoking effects through contrast sensitivity function (CSF), a rigorous procedure that measures the spatial vision. Data were recorded from 48 participants, a group of non-smokers (n = 16), a group of chronic and heavy cigarette smokers (n = 16) and deprived smokers (n = 16); age range 20–45 years. Sinewave gratings with spatial frequencies ranging from 0.25 to 20 cycles per degree were used. All subjects were free from any neurological disorder, identifiable ocular disease and had normal acuity. No abnormalities were detected in the fundoscopic examination and in the optical coherence tomography exam. Contrary to expectations, performance on CSF differed between groups. Both smokers and deprived smokers presented a loss of contrast sensitivity compared to non-smokers. Post-hoc analyses suggest that deprived smokers were less sensitive at all spatial frequencies. These results suggest that not only chronic exposure to cigarette compounds but also withdrawal from nicotine affected spatial vision. This highlights the importance of understanding diffuse effects of smoking compounds on visual spatial processing.

## Introduction

Cigarette smoking consists of numerous compounds harmful to health^1^. Data from the World Health Organization (WHO) hypothesize that by 2030, cigarettes could kill nearly 9 million people a year around the world^2, 3^.

Nicotine, one psychoactive compound of cigarette smoke, is an alkaloid that binds and activates nicotinic acetylcholine receptors (nAChRs)^4^. There are nAChRs receptors in functional units of visual processing such as retinal cells, lateral geniculate nucleus, and primary visual cortex of numerous species^5, 6^.

The imbalance in the neurotransmission of acetylcholine, dopamine and glutamate as well as impairments in the receptors functioning or conformation alter visual information processing^7, 8^. Studies have shown that nicotinic agonists (nicotine, for example) affect the release of these neurotransmitters^4, 9^.

Whereas there is a lack of studies about smoking effects on spatial vision, the existing data are a little controversial and highlight the need of a rigorous testing procedure that measures spatial vision^10–12^. In addition, there is a need to understand which (if any) mechanisms involve cigarette toxicity in sensory integration. Therefore, we first need to understand how smoking can change early visual processing.

Contrast sensitivity function (CSF), an important measure of visual function and the first analysis performed by the visual cortex^13, 14^, may be used to assess the performance of the visual system. Luminance contrast differences, which are perceived as lightness differences, can be identified and quantified through spatial frequency responses^13, 14^.

Some benefits from the use of acute nicotine were reported in some studies that investigated cognitive functions such as memory and attention reported. However, it is interesting to point out that these same cognitive functions are affected due to the nicotine chronic intake in the form of tobacco addiction. Since vision may be a gateway to cognition^15^, understanding changes in primary visual processing may help promote smoke cessation strategies.

Based on this information, the purpose of the present study was to investigate the influence of chronic heavy smoking and smoking abstinence on spatial vision.

## Results

In this study, CSF is expressed as the reciprocal logarithm of the threshold contrast. There was a statistically significant difference in CSF among all groups, χ²(2) = 14.66, *p* < 0.001. Contrast sensitivity peak values occurred at spatial frequencies of 2.5 cpd and 5 cpd with decays at low (0.2 cpd) and high (10 and 20 cpd) spatial frequencies.

### Psychophysical measurements

**Figure 1:**
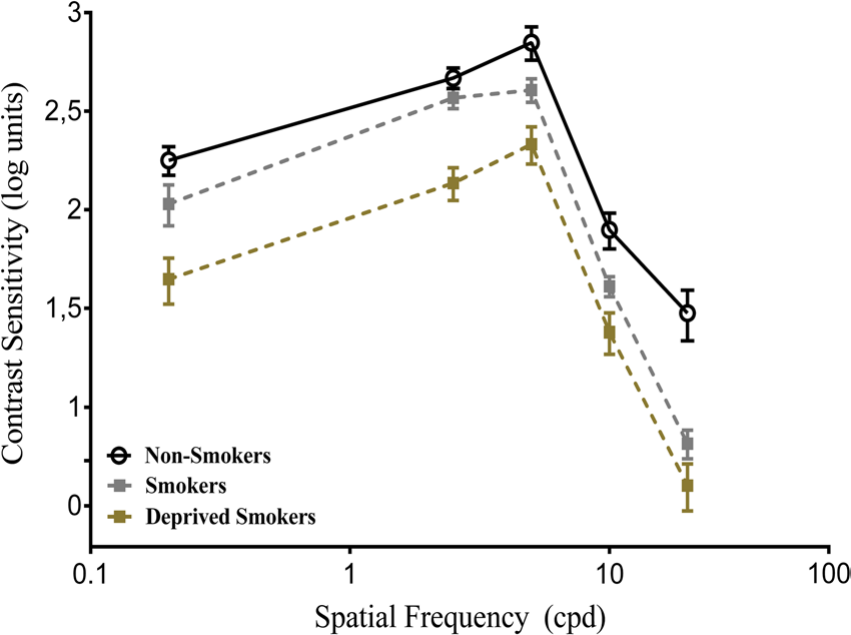
Contrast sensitivity curves as a function of spatial frequency (cpd) for non-smokers, smokers and deprived smokers. Each data point represents the sensitivity (reciprocal of contrast threshold) and error bars represents the standard deviation (SD) of the median sensitivity based on 1000 bootstrap resamplings. CSF is plotted in logarithmic units.

The results of the psychophysical contrast sensitivity measurements are shown in Fig. 1. The non-smokers group had significantly higher sensitivity than deprived smokers at all spatial frequencies, 0.2 cpd (*U* = 91, *p* = 0.001, *r* = −0.70), 2.5 cpd (*U* = 92, *p* = 0.001, *r* = −0.69), 5.0 cpd (*U* = 94, *p* = 0.003, *r* = −0.66), 10.0 cpd (*U* = 81, *p* < 0.001, *r* = −0.82) and 20.0 cpd (*U* = 96, *p* = 0.002, *r* = −0.64). Thus, non-smokers needed 0.37, 0.35, 0.34, 0.43 and 0.32 log units less contrast for low, mid and high spatial frequencies, respectively.

The CSF of deprived smokers was significantly lower than smokers were at spatial frequencies of 0.2 cpd (*U* = 110, *p* = 0.019, *r* = −0.48), 2.5 cpd (*U* = 109, *p* = 0.018, *r* = −0.48), 5.0 cpd (*U* = 107, *p* = 0.013, *r* = −0.51), and 10.0 cpd (*U* = 110, *p* = 0.021, *r* = −0.47) except for 20.0 cpd (*U* = 124, *p* = 0.133). In general, deprived smokers needed 0.24, 0.24, 0.25 and 0.23 log units more contrast than smokers did for 0.2 cpd, 2.5 cpd, 5.0 cpd and 10.0 cpd, respectively.

Briefly, post-hoc test showed statistically significant differences between non-smokers and smokers at spatial frequencies of 5.0 cpd (*U* = 116, *p* = 0.025, *r* = −0.46), 10.0 cpd (*U* = 117, *p* = 0.030, *r* = −0.44) and 20.0 cpd (*U* = 97, *p* = 0.002, *r* = −63). There were no differences between 0.2 cpd (*U* = 126, *p* = 0.145) and 2.5 cpd (*U* = 131, *p* = 0.455).

### Correlation analysis of demographics data

**Figure 2:**
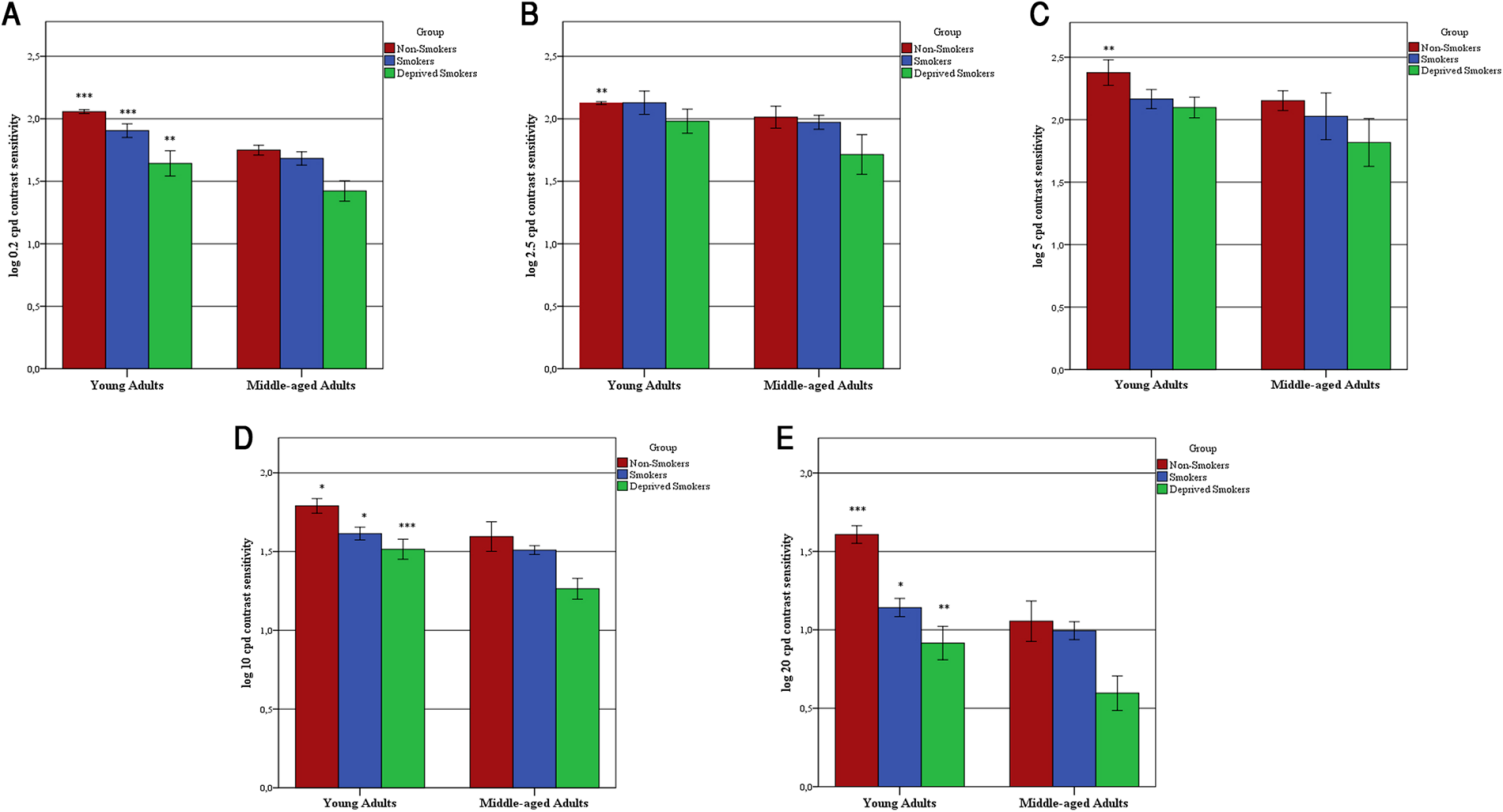
Contrast sensitivity bars as a function of age groups for non-smokers, smokers and deprived smokers at each spatial frequency. Color bars represents the sensitivity (reciprocal of contrast threshold) for each spatial frequency. Error bars represents the standard deviation (*SD*) of the mean sensitivity. ***p < 0.001, **p < 0.01, *p < 0.05.

There is no relationship between CSF and gender (chi-square = 36, *df* = 33, *p* > 0.05) and education years [rho = 0.080, *p* = 0.578]. On the other hand, there was a strong, negative correlation between age and CSF data (*p* < 0.05) at low (0.2 and 2.5 cpd) and high spatial frequencies (20 cpd). Middle-aged adults had poorer CSF at 0.2 cpd (rho = −0.597, *p* < 0.001), 2.5 cpd (rho = −0.621, *p* < 0.001), 10.0 cpd (rho = −0.343, *p* = 0.045) and 20 cpd (rho = −0.434, *p* = 0.008), except for 5.0 cpd (rho = −0.222, *p* = 0.193) (Fig. 2). A spearman correlation showed no correlation between FTND and CSF (*p* > 0.050).

## Discussion

The data indicated that smokers, as a whole, demonstrated reduced CSF when compared to non-smokers. High spatial frequencies deficits were the most frequent type of divergences observed among smokers group, at about 0.25 log units of differences between non-smokers and deprived smokers groups. Despite of a lower sensitivity, the relative difference between smokers and non-smokers was minimal if we take as a parameter the CSF of deprived smokers group (Fig. 1).

It is known that there are numerous compounds in the cigarette that, per se, are harmful such as carbon monoxide, toluene, tar, nickel and many others. Thus, the intent of the present study was not to specifically evaluate the effect of nicotine, one of the components of the cigarette, but to observe and investigate the effects of cigarette smoking in visual spatial processing. In general, the results indicated that smokers presented less sensitivity when compared to the non-smokers group. In agreement with some studies^16–18^ and in disagreement with other studies^12, 19, 20^ that showed effects of cigarette and nicotine on visual processing, these data point to a new investigation direction.

The results do not suggest the existence of selective changes in spatial channels of visual processing^12, 20^, but support the idea that tobacco constituents play a role as addiction chemical modulators and visual sensitivity through dopamine (DA), GABA and glutamate neurotransmission deficits^21–26^.

Nicotine, for example, a tobacco psychoactive component, enhances DA release through a dynamic balance of activation and desensitization of nAChRs located mainly in the ventral tegmental area (VTA) and in the striatum^9, 27^. Since there are also nAChRs and DA receptors on the retina, it is not hard to understand that acute use of nicotine would enhance cognition at some points^28–30^, since nicotine’s effects on multiple neurotransmitters are thought to contribute to cognitive performance^27^.

In this case, we did not observe a large reduction of CSF among smokers, but also did not observe improvements. So, what would be the relation between chronic smoking and visual processing? The answer may lie in desensitization, which is one of many changes caused my addiction^31^. Besides, chronic nicotine exposure leads to nAChRs desensitization through brain upregulation^4, 32^. Another property of tobacco, specifically from nicotine, is that the more exposure, the greater the need for it activate the receptors, which changes affinity and response properties of the nAChRs^33, 34^. Considering that nicotine-enhancing effects decrease over time and remain unchanged after chronic exposure, this may explain not only the lower CSF but also the similarity between smokers and non-smokers in our data (Fig. 1).

On the one hand, smokers group needed nearly two times more contrast to detect high frequencies when compared to non-smokers (*p* < 0.001, *r* = −0.60). When we compare with deprived smokers, they were less sensitive in order of 0.5 log units at low (*p* < 0.005) and mid spatial frequencies (*p* < 0.007) (Fig. 1). Thus, the high spatial frequencies were the most affected comparing smokers and deprived smokers to non-smokers, and the least affected when comparing smokers and non-smokers. As stated, these results do not support the idea of channel selectivity deficits, despite the existence of nAChRs in the primary visual cortex^35^. However, we hypothesize the existence of a diffuse deficit in visual processing, which may include magno- and parvocelular pathways, since we used a photopic condition and observed significant changes at high spatial frequencies^8, 11^.

On the other hand, deprived smokers showed a large reduction in the CSF (Fig. 1). As expected, the reduction of CSF occurred at all frequencies, when comparing with non-smokers (*p* < 0.001). However, we expected that differences between smokers and deprived smokers would be minimal, without statistically significant differences. Our data showed that deprived smokers were more sensitive for almost all spatial frequencies (*p* < 0.05), except 20.0 cpd. This can be explained by withdrawal effect (≤24 h) which induces a hypofunctional effect of dopamine release^36, 37^, reflecting at both visual processing^38–40^ and brain reward function^41^. Tobacco deprivation, even in a short period, play a role affecting motivation and attention in responding to the test stimuli^42, 43^. Therefore, we suggest the existence of another factor within tobacco deprivation: attentional bias.

In order to detect environmental stimuli, visual attention is a key mechanism the brain uses. Some psychophysical studies indicated that directing attention to a certain location has an influence on spatiotemporal aspects of visual processing^44–46^. Considering attention and motivation as functions affected with tobacco deprivation, they would then play an important role in the CSF decrease, even comparing against smokers group. Besides, this may explain the larger decrease at low spatial frequencies (*p* < 0.001), once that deprivation stimulates the amygdala and activates the magnocellular system which, in turn is specialized in providing low spatial frequency information^47^.

Although smoking behavior is slightly different between men and women^48^, there were no gender differences in our data. We observed a strong negative correlation between age and CSF, being that the middle-aged adults had a worse CSF than young adults (Fig. 2). This same pattern was found in other studies using psychophysics^49–51^. It is hypothesized that the normal aging affects both magno- and parvocelular pathways in humans and the parvocellular is the most largely affected. Our data pointed out that high frequencies were the most impaired between groups (Fig. 2). Thus, it is consistent with Flaubert’s theory of visual perception and aging^52^.

Even though there are a limited number of studies about smoking and visual processing, specifically about spatial vision, our limitations should be considered. First, this was an observational study, and such a design does not permit evaluation of the physiological, mechanistic, or genetic associations between smoking and spatial vision *per se*. Second, even the use of a classic, reliable and rigorous testing procedure such as CSF may be unable to assess all aspects of visual function important during smoking abstinence. Therefore, we recommend the combined use of multiple methods (e.g., physiological, behavioral and imaging) with psychophysics to facilitate the understanding of the mechanisms involved. Third, we evaluated tobacco as a whole, not only the nicotine effects^19, 20^, which brings us the idea of further studies, using nicotine gum and the same paradigm used here.

Clearly, further work is needed but this study highlights the relation between smoking and spatial visual processing. We suggest that cigarette components may affect vision more than nicotine separately - which even helps in disorders such as Schizophrenia and Parkinson’s Disease^53^. Indeed, tobacco components may increase free radical that would cause macular degeneration along with the action of ischemia. Besides, these mechanisms affect color vision^19, 21, 54^, which is our future research target.

## Methods

### Participants

This research followed the ethical principles from the Declaration of Helsinki and was approved by the Committee of Ethics in Research of the Health Sciences Center of Federal University of Paraiba (CAAE: 60944816.3.0000.5188). Written informed consent was obtained from all participants.

In this study, 16 non-smokers (mean age = 34.1 years; *SD* = 7.9; 8 male), 16 heavy cigarette smokers (mean age = 34.4 years; *SD* = 7.3; 8 male) and 16 deprived smokers (mean age = 30.3 years; *SD* = 5.1; 8 male), from 25 to 45 years, who were working as staff or were students at Federal University of Paraiba, were recruited through newspaper advertisements. Exclusion criteria were: (I) older than 45 years (since the effects of the human visual system maturation or aging could superestimate the results^51^), (II) current history of neurological disorder, (III) a history of head trauma, (IV) history of contact with substances such as solvents, (V) met criteria for a lifetime diagnosis of substance abuse or dependence other than nicotine dependence (for smokers groups), (VI) drinking more than 10 alcoholic drinks per week, or (VII) current use of medications that may affect visual processing and cognition. In addition, subjects were required to have a good ocular health: no abnormalities were detected in the fundoscopic examination and in the optical coherence tomography exam. All observers were screened for color blindness using Ishihara’s^55^ tests for color deficiency and had normal or corrected-to-normal vision as determined by a visual acuity of at least 20/20.

Smokers reported a smoking history of at least 8 years, currently smoked more than 20 cigarettes/day and had a score >5 on the Fagerström Test for Nicotine Dependence (FTND)^56^. Smokers and deprived smokers began smoking on average at the age of 16.5 years (*SD* = 1.05) and had been smoking on average for 17 years (*SD* = 4.05). Smokers were allowed to smoke until the beginning of the experiment. An abstinence period of 12 hours was chosen based on previous studies. Non-smokers had never smoked a cigarette. To assess subjective craving for a cigarette (a form of evaluation of abstinence), participants provided self-reporting and completed psychometric measures such as Program to Aid Smokers (PAS)^28^ comfort scale and the brief version of the Questionnaire of Smoking Urges (QSU-B)^57^. There were no statistical differences between depression and anxiety symptoms pre- and post-experiment, as measured by Hamilton Scale for Depression and Hamilton Anxiety Rating Scale.

The groups did not differ in age, *F* (2, 47) = 0.17, *p* = 0.98, or ratio of males to females (Pearson’s Chi Square Value = 1.9, ns). There was also no difference in FTND scores (*t*(1) = 2.35, *p* = 0.98), age at first cigarette use (*t*(1) = 5.71, *p* = 0.99), and years of cigarette use (*t*(1) = 1.63, *p* = 0.93) between the two groups of smokers who took part in this experiment (Table 1).

**Table 1:** Sample characteristics (N = 48).

Variables	Non-Smokers (n = 16)	Smokers (n = 16)	Deprived Smokers (n = 16)	T-test (*p-value*)
Gender				
Male	8	8	8	—
Female	8	8	8	—
Age				
Young Adults	8	9	8	—
Middle-aged	8	7	8	—
Adults				
Education Level				
High School	5	4	4	—
College	11	12	12	—
Cigarette use				
Age at first use	—	17 ± 1.4	16 ± 0.7	5.71 (*0.99*)
Years of use	—	16 ± 4.9	18 ± 3.2	1.63 (*0.93*)
FTND	—	7 ± 1.7	8 ± 0.5	2.35 (*0.98*)
PAS Comfort Scale				
Before experiment	—	19.50 ± 2.3	18.44 ± 2.1*	0.81 (*0.93*)
After experiment	—	19.25 ± 1.4	10.63 ± 1.9	8.14 (*0.000*)
QSU-B				
Before experiment	—	28.81 ± 7.6	29.69 ± 6.3	−3.53 (*0.72*)
After experiment	—	26.63 ± 3.7	53.4 ± 5.1*	−17.01 (*0.000*)

*Statistically significant difference at pairwise comparison between deprived smokers (*p* < 0.001).

### Stimuli and Apparatus

Stimuli were presented on a 19-inch LG CRT monitor with 1024 × 786 resolution and a rate of 100 Hz. Stimuli were generated using a VSG 2/5 video card (Cambridge Research Systems Ltd., Rochester, Kent, UK), which was run on a microcomputer Precision T3500 with W3530 graphics card. The average luminance was 50 cd/m². All procedures were performed in a room at 26 ± 1 °C, with the walls covered in grey for better control of luminance during the experiments. Measurements were performed with binocular vision at a distance of 150 cm from the monitor screen. Monitor luminance and chromatic calibrations were performed with a ColorCAL MKII photometer (Cambridge Research Systems).

The visual contrast sensitivity measurements were taken through Metropsis software (Cambridge Research Systems Ltd., Rochester, Kent, UK). This software provides a CSF clinical evaluation. The Metropsis vision-testing suite provides precise, repeatable, psychophysical threshold measurements for general research applications.

Similar to Andrade *et al*.^58^, we used the same procedure where Metropsis was the software responsible for generating vertical sinewave gratings. Spatial frequencies of 0.2, 2.5, 5.0, 10.0 and 20.0 cpd, with an initial phase of 180°, were responsible for the luminance contrast sensitivity measurements (for details, see ref. 11). Stimuli consisted in equiluminant gratings with dimensions of 5 degrees of visual angle, being presented in the monitor at 2.5 degrees from the cross-shaped fixation point.

### Procedure

Before scheduling the experiments, participants were advised about the research procedures and perspectives. The division of smokers and deprived smokers groups occurred randomly. Along with the recommendations (e.g., abstinence from caffeine or psychoactive substances), information on cigarette deprivation was given instantaneously. To schedule participation in the study, deprived smokers were asked to avoid the use of cigarettes 12 hours before the beginning of the experiments.

Contrast sensitivity measurements were performed using the two-alternative forced choice (2-AFC) method, and the subjects’ task was to identify, using a remote control response box, whether the stimulus was presented at the left or right side of the screen. Subjects were instructed to maintain fixation on a small black fixation cross in the center of the display monitor. The order of frequencies tested was randomized within a session.

A three-down one-up logarithmic staircase with dynamic steps was used, tracking performance at 75% accuracy. Initially, the contrast values appeared in suprathreshold level (which was expected that participants had a series of correct answers) to reach the staircase criteria of three consecutive correct responses and an error. Thus, after three consecutive correct responses contrast decreased 0.7 dB and increased by 1.0 dB after every incorrect response. Stimuli had exposure time of 600 ms with between-intervals of 300 ms. This process took place throughout the experiment.

To obtain the reversals for each grating, an average of 150–250 trials (depending on the participant) were presented. The session ended after eight response reversals that were recorded for each grating tested. The thresholds were calculated averaging the contrast values of the response reversals for each grating, and contrast sensitivity was estimated as the reciprocal of the threshold values. Higher contrast sensitivity values mean that the participant presents higher sensitivity to the spatial frequency evaluated in the test (for procedure details, refer to refs 58 and 59).

### Statistics

The distributions for each group were compared with Shapiro-Wilk. Both groups showed non-normal distribution, thus non-parametric statistical methods were used to analyze the data. For group comparisons, the non-parametric univariate analysis was used, with pairwise comparisons by Mann-Whitney *U* test. Spearman’s rank correlation coefficients (rho) were conducted to assess the relation between outcomes of color discrimination data and biosociodemographic variables such as age, gender and education level.

The effect size (*r*) estimation was used from the conversion of z-score^60^. Each data point represents the medians and errors bars represent standard deviations (SD) of the median based on 1000 bootstrap resamplings. Bonferroni correction was the method of adjusting *p*-value that was used.
